# Reasons for using and efficacy of raltegravir in salvage regimens without protease inhibitors in clinical practice

**DOI:** 10.1186/1758-2652-13-S4-P35

**Published:** 2010-11-08

**Authors:** JM Llibre, E Martínez, P Barreiro, R Escrig, E Ribera, M Cervantes, A Imaz, F Gutierrez, H Knobel, A Ornelas, FX Zamora, B Clotet

**Affiliations:** 1 University Hospital Germans Trias, Lluita contra la SIDA Fndn, Badalona, Spain; 2 Hospital Clinic IDIBAPS, Barcelona, Spain; 3 Hospital Carlos III, Madrid, Spain; 4 Lluita contra la SIDA Fndn, Badalona, Spain; 5 Hospital Vall d'Hebró, Barcelona, Spain; 6 Hospitals Parc Tauli, Sabadell, Spain; 7 Hospital de Bellvitge, L'Hospitalet de Llobregat, Spain; 8 Hospital Universitario de Elche, Elche, Spain; 9 Hospital del Mar, Barcelona, Spain; 10 Hospital La Paz, HIV Unit, Madrid, Spain

## Purpose of the study

The efficacy of raltegravir (RAL) in salvage regimens without protease inhibitors (PI) has not been evaluated in randomized trials, and information about its efficacy and reasons for its initiation in cohort studies has received scant attention. In some particular scenarios physicians may be forced to use RAL without PI due to advanced protease resistance, toxicity or patient refusal.

## Methods

Systematic multicenter search of databases in University-affiliated hospitals in Spain to identify all pre-treated patients with limited options due to resistance or intolerance to multiple antiretrovirals, starting a regimen including raltegravir and not a PI for any given reason, with a baseline plasma HIV-1 viral load (VL) >500 copies/mL. Primary endpoint: proportion achieving a VL<50 c/mL at 48 weeks.

## Results

We identified 55 patients, 69% male, with a median age of 45,8 y., 40% IVDU, 45% with chronic hepatitis C, 40% in stage CDC C. Fourteen (25%) were diagnosed of dyslipidemia. 19/20 (95%) patients with results available had a CCR5 tropism. The main reasons for initiating a regimen with RAL and without PI were advanced protease resistance (44%), toxicity (15%), pharmacokinetic interactions (11%), and patient refusal (7%). The most frequently used drugs on board were tenofovir in 43 (78%) patients, maraviroc in 16 (29%) patients, and etravirine in 11 (20%). Eight (14%) patients did not use any nucleoside analogue, all of them due to complete resistance or toxicity. At 48 weeks, 66% of them had an HIV-1 VL < 50 c/mL. There was a significant increase in the median CD4 cell count from 257 cells/<micro>L at baseline to 415 cells/µL at 48 weeks (p=0.01), with only 19% of patients remaining with <200 CD4 cells/µL at 48 weeks. Virological failure was documented in 4 (7%) patients. There were no unexpected adverse events related to RAL when used without PI.

## Conclusions

Salvage regimens including RAL but not PI may be used in selected patients mainly due to advanced protease resistance, toxicity, pharmacokinetic interactions or patient refusal to PI. The efficacy of raltegravir when used with a background regimen without a protease inhibitor is high, but the inclusion of 2 further active drugs must be strongly pursued. This cohort analysis supports further study of RAL with novel combinations in this difficult-to-treat population.

